# Development of Flexible Biceps Tremors Sensing Chip of PVDF Fibers with Nano-Silver Particles by Near-Field Electrospinning

**DOI:** 10.3390/polym14020331

**Published:** 2022-01-14

**Authors:** Chung-Kun Yen, Karishma Dutt, Yu-Syuan Yao, Wen-Jeng Wu, Yow-Ling Shiue, Cheng-Tang Pan, Chi-Wen Chen, Wen-Fan Chen

**Affiliations:** 1 Department of Mechanical and Electro-Mechanical Engineering, National Sun Yat-sen University, Kaohsiung 80424, Taiwan; alden0113@gmail.com (C.-K.Y.); karishma2019@mem.nsysu.edu.tw (K.D.); pan@mem.nsysu.edu.tw (C.-T.P.); 2 Department of Mechanical and Automation Engineering, I-Shou University, Kaohsiung 84001, Taiwan; 3 Institute of Biomedical Sciences, National Sun Yat-sen University, Kaohsiung 80424, Taiwan; as0917520936@gmail.com (Y.-S.Y.); shirley@imst.nsysu.edu.tw (Y.-L.S.); 4 Graduate Institute of Clinical Medicine, College of Medicine, Kaohsiung Medical University, Kaohsiung 80756, Taiwan; wejewu@kmu.edu.tw; 5 Department of Urology, School of Medicine, College of Medicine, Kaohsiung Medical University, Kaohsiung 80756, Taiwan; 6 Department of Urology, Kaohsiung Municipal Ta-Tung Hospital, Kaohsiung 80145, Taiwan; 7 Center for Stem Cell Research, Kaohsiung Medical University, Kaohsiung 80756, Taiwan; 8 Institute of Precision Medicine, National Sun Yat-sen University, Kaohsiung 80424, Taiwan; 9 Department of Orthopaedics, Kaohsiung Armed Forces General Hospital, Kaohsiung 80284, Taiwan; 10 Institute of Medical Science and Technology, National Sun Yat-sen University, Kaohsiung 80424, Taiwan

**Keywords:** piezoelectric fiber, near-field electrospinning, AgNO_3_, PVDF, sensing element, fasciculation

## Abstract

Polyvinylidene fluoride (PVDF) and AgNO_3_/PVDF composite piezoelectric fibers were prepared using near-field electrospinning technology. The prepared fibers are attached to the electrode sheet and encapsulated with polydimethylsiloxane to create an energy acquisition device and further fabricated into a dynamic sensing element. The addition of AgNO_3_ significantly increased the conductivity of the solution from 40.33 μS/cm to 883.59 μS/cm, which in turn made the fiber drawing condition smoother with the increase of high voltage electric field and reduced the fiber wire diameter size from 0.37 μm to 0.23 μm. The tapping test shows that the voltage signal can reach ~0.9 V at a frequency of 7 Hz, and the energy conversion efficiency is twice that of the PVDF output voltage. The addition of AgNO_3_ effectively enhances the molecular bonding ability, which effectively increases the piezoelectric constants of PVDF piezoelectric fibers. When the human body is exercised for a long period of time and the body is overloaded, the biceps muscle is found to produce 8 to 16 tremors/second through five arm flexion movements. The voltage output of the flexible dynamic soft sensor is between 0.7–0.9 V and shows an orderly alternating current waveform of voltage signals. The sensor can be used to detect muscle tremors after high-intensity training and to obtain advance information about changes in the symptoms of fasciculation, allowing for more accurate diagnosis and treatment.

## 1. Introduction

From the industrial field to the smart home, sensors have changed the habits of people in the past, so these systems have become indispensable wearable devices in people’s lives. In the field of smart wear, sensors can be used to distinguish direction, navigation, and motion assistance, and they can measure, record, and analyze human activities anytime and anywhere, and even further analyze sleep quality. Furthermore, with the help of wearable field sensor technology, it can further alert and monitor health conditions and improve diagnostic accuracy in the medical field. There are several types of pressure sensors on the market today, including capacitive sensors [1,2], resistive sensors [3,4], piezoelectric sensors [5,6], and piezoresistive sensors [7,8]. These sensors provide an electrical signal output proportional to the measured pressure. In terms of sensor performance, the key specifications required for pressure sensors include linearity, hysteresis, temperature sensitivity, sensing size and pressure range [9].

Of all the materials in the world, only about 30 percent can exhibit piezoelectricity. Despite the fact that a wide variety of materials have this property, there are only a few materials that can find more effective applications. Piezoelectric materials can be broadly classified into the following categories [10]; single crystal [11], polycrystalline [12], relaxor ferroelectric [13], polymer [14], and piezoelectric paper [15]. Polyvinylidene fluoride (PVDF) and its derivatives are the most widely used materials in flexible mechanical and electrical equipment in recent years, with high flexibility, high sensitivity, high ductility and certain piezoelectric coefficient. This material can be used in wearable functional devices, chemical sensors, biosensors, and flexible actuator devices for flexible microelectromechanical devices [16]. PVDF is one of the most attractive polymer materials for mechanical energy harvesting applications, with properties such as high piezoelectric coefficient (d33 = 49.6 pm/V), excellent stability and the required flexibility for nano-generator applications. Among the methods used to fabricate PVDF nanofibers, electrospinning is considered as one of the most efficient methods due to its simplicity and low cost, and the voltage applied during the synthesis process helps to obtain the desired PVDF phase (β-phase). During the electrostatic spinning process, the piezoelectric properties are enhanced by rearranging the α-helix of the peptide and by transforming the nonpolar α-phase of the PVDF solution into a polar β-phase, which is responsible for the piezoelectric properties [17]. Further, this method may generate a large amount of voltage during mechanical stress, strain and bending [18].

Kawai et al. discovered the piezoelectric properties in PVDF by subjecting the polymer to mechanical stretching and electric fields in 1969 [19,20]. PVDF has five known phases, namely α, β, γ, δ and ε. The polar crystal structure (β, γ, δ) defines the electroactivity in which all dipoles are parallel, thus giving rise to a non-zero dipole moment [21]. Due to the excellent piezoelectric, thermoelectric and ferroelectric properties, the β-phase is considered as an essential crystal form [20]. In the case of β-phase, all dipole moments point in the same direction and show the highest piezoelectric response [22]. The electrical activity of PVDF depends mainly on the β-phase content. There are different methods to increase the β-phase content, such as mechanical stretching, use of polar solvents to dissolve and recrystallize PVDF, electropolarization, and addition of fillers [23]. In the hollow cylindrical near-field electrospinning (HCNFES) process, in-situ polarization and high uniaxial stretching are performed on the polymer jet, which aligns the dipoles in the PVDF crystals and promotes the formation of polar β-crystalline phases within the fibers [24].

In this study, the proposed near-field electrostatic spinning technique is applied to the development of flexible soft dynamic sensing elements with Ag nanoparticles of PVDF piezoelectric fibers. The piezoelectric fibers are produced by adding Ag nanoparticles to the PVDF polymer solution through a near-field electrostatic spinning process with roller collection to produce orderly and large-area piezoelectric fibers. The process of electrospinning is carried out by the weight percentage of the precursor solution and the electrical conductivity at the optimum electrospinning process parameters. Finally, a combination of interdigitated electrodes (IDT) and flexible PET substrates encapsulated in polydimethylsiloxane (PDMS), a flexible dynamic soft-sensitive device can be applied to smart wears to detect muscle tremors after high-intensity training and to obtain advance information on fasciculation.

## 2. Materials and Method

**Figure 1:**
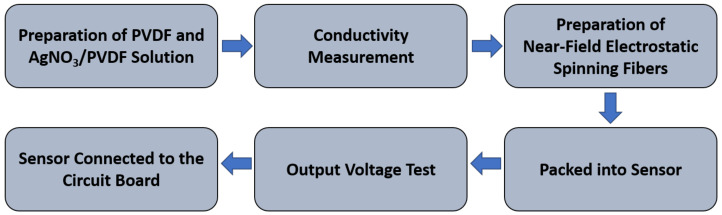
Overall experiment flowchart.

An electrostatic spinning process with rollers was used to collect sequential piezoelectric fibers. The PVDF and AgNO_3_/PVDF blended solutions (Sigma–Aldrich, St. Louis, MO, USA) were used to find the appropriate process parameters, including the electric field strength, the push rate of the solution, and the tangent speed of the roller collector, for electrospinning. The fibers produced after the electrospinning process are further used to fabricate the harvest device. The device does not require additional power supply, as the device itself is a self-powered PVDF piezoelectric harvester. The kinetic sensor is further developed by using the circuit board. The experimental flow is shown in Figure 1.

### 2.1. Preparation of PVDF Solution

The preparation of PVDF solution can be divided into two parts, Solution A and Solution B. The proportions are shown in Table 1. Solution A was prepared by adding 0.9 g of PVDF powder into a scintillation vial and then adding 2.5 g of acetone solution for mixing. Mix the solution with a magnet stirrer (speed = 400 rpm) on a heated mixer at room temperature for 30 min to disperse the powder evenly in the acetone solvent. After stirring, the solution appeared milky white. Solution B was prepared by adding 0.2 g of surfactant to the scintillation vial and mixing with 2.5 g of dimethyl sulfoxide (DMSO). Stir for 30 min at room temperature using a magnet stirrer (speed = 400 rpm), at which point Solution B appeared light brown. The purpose of adding the surfactant is to reduce the surface tension of the electrospinning solution in the air so that the solution can break through the surface tension more easily during the electrospinning process and obtain continuous piezoelectric fibers. Then, pour solution A into solution B and stir at 400 rpm for 30 min at room temperature. After stirring, the solution was translucent and milky white. Finally, the solution was left to stand for 30 min to reduce the air bubble residue in the solution. For the preparation of AgNO_3_/PVDF composite solution, the experimental procedure is similar to the preparation of pure PVDF solution; the only difference is the addition of 6% AgNO_3_. In the process of near-field electrostatic spinning, the addition of 6% AgNO_3_ allows the PVDF solution to accumulate charge rapidly without causing a short circuit due to high electrical conductivity. If less than 6% AgNO_3_ is added, the adhesion and electrical conductivity between the liquid molecules would be lower and the larger fiber diameter would be formed. If AgNO_3_ is added more than 6%, although it can reduce the viscosity of PVDF and increase the electrical conductivity, the solution conductivity would be too high and further cause short circuit during the electrospinning process. Hence, 6% AgNO_3_ was added to the PVDF solution in this study. Further, the previous work [25] have been confirmed that Ag ions have been cooperated in PVDF in the AgNO3/PVDF fiber.

### 2.2. Near-Field Electrostatic Spinning Process

**Figure 2:**
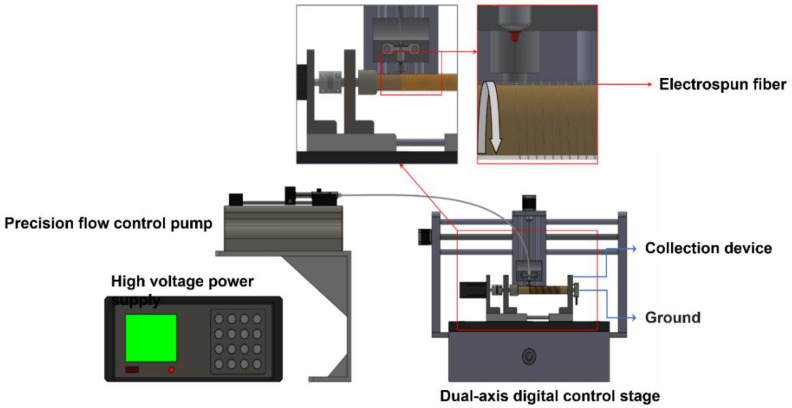
Schematic diagram of near-field electrostatic spinning equipment.

The polymer solution is poured into a push-in cartridge and held in place on a precision metering pump (World Precision Instruments, Sarasota, FL, USA). The stainless-steel dispensing needle of the syringe is connected to a high-voltage field (positive) power supply as the nozzle for near-field electrostatic spinning, and the grounded part is composed of a glass tube (20 mm outer diameter, 0.50 mm thickness) collecting piezoelectric fibers, a direct current (DC) motor, and a negative high-voltage power supply (negative). A copper tape of about 0.10 mm thickness is attached to the inside of the glass tube to facilitate the electric field and contact with the ground (negative electrode). When a high voltage is supplied to the needle, an electric field is formed between the needle and the glass tube, causing the solution at the front of the needle to be attracted by the static force of the applied electric field. At this point, the surface of the droplet will accumulate charges, and the charges of the same electrical properties will repel each other and form a Taylor cone. When the static force of the charge accumulated by the Taylor cone is greater than the surface tension of the droplet, it will be ejected into a filament through the droplet on the Taylor cone. If the bonding force between the liquid molecules is high enough, the ejected liquid will not split apart, but will form a stream (i.e., fibers formation). In the process of fiber formation, the heat conduction increases due to the high electric field, which effectively improves the evaporation of the solvent. The computer-controlled moving platform allows the needle to move laterally to collect a large area of fibers in an orderly manner with a specific stretch direction. The needle and collection device used in this study collects fibers in a vertical manner, which helps to break the surface tension due to gravity. The needle model is 24G (aperture ~0.381mm) with 16 kV voltage (field strength 1.6 × 10^7^ V/m), 1 mm between the needle and roller collection device, and 2 mm/s movement rate of the XY dual-axis digital control stage. Using the precision flow control pump to control the flow rate of the solution, the rate of 0.01 mL/min is fixed to make the fiber more smoothly without interruption. The schematic diagram of the near-field electrostatic spinning equipment is shown in Figure 2.

### 2.3. Sensor Package

**Figure 3:**
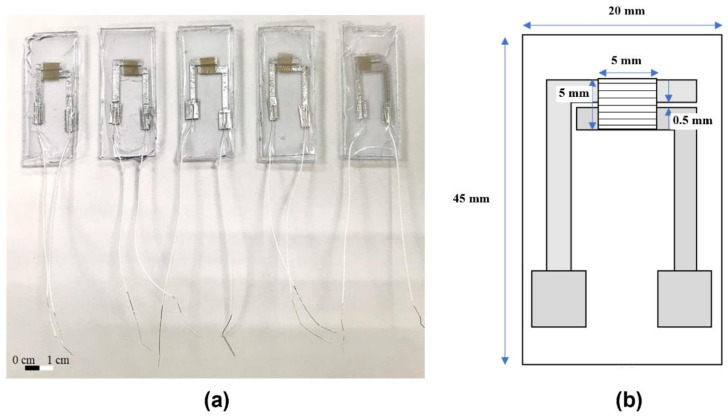
(**a**) Finished package of the sensor and (**b**) schematic diagram of the package dimensions (about 1500 fibers are laid on, the outermost layer of the package is made of PDMS, the electrodes are glued on with PET as the base plate, and the electrode material is aluminum).

A flexible sensor was prepared using Polydimethylsiloxane (PDMS). The type of PDMS used is 184, and the solution was divided into A and B agents (produced by SIL-MORE). Agent A is epoxy resin and agent B is curing agent. Mixed the A agent with the B agent using the ratio of 10:1 and added it to the beaker covered with plastic wrap, placed it on a blender and mixed for 30 min. The electrodes were glued to the PET backing (1.9 mm × 4.4 mm), where the gap is 1 mm. A small amount of PDMS was then poured into the mold to hold the base plate to prevent the fiber from drifting after being placed on it. Afterwards, the molds were placed in TARSONS^®^ PP/PC desiccator vacuum (Tarsons, Kolkata, India) for 15 min to eliminate air bubbles. The mold was placed with polyethylene terephthalate (PET) coated with silver wire on the heater and heated it at 100 °C for 15 min. After curing, the fiber was put on, the remaining PDMS was poured into the package, and vacuumed for 15 min. After that, heated on the heating plate for 15 min as the previous steps. When finished, the samples were removed using tweezers. The AgNO_3_-added fibers require additional UV light to excite silver ions, and the final products are shown in Figure 3.

### 2.4. Characterization

The conductivity of PVDF and AgNO_3_/PVDF electrospinning solutions is measured using a CLEAN CON30 conductivity meter (Twinno, New Taipei, Taiwan). When the electrical conductivity is higher, the amount of charge carried in the solution will also increase, making it easier for the droplets to break through the surface tension. If the electrical conductivity is low, the solution is not completely stretched, and the homogeneity of the fiber will be poor. Scanning electron microscope (SEM; JEOL JSM-63800, Akishima, Japan) was used to observe the morphology of the fibers. Fourier transform infrared spectrometer (FTIR, Nicolet iS50, Thermo Fisher, WA, USA) was used to observe the features and wave numbers of absorption peaks to identify the functional group types and structures of molecules.

### 2.5. Electrical Measurements

**Figure 4:**
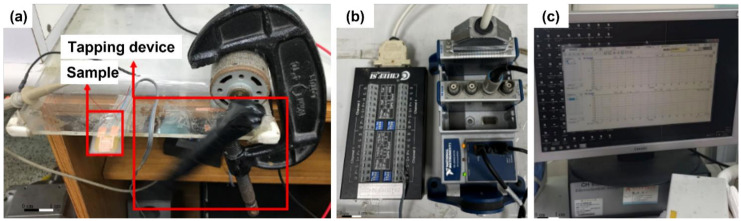
Electrical measurement equipment: (**a**) actual tapping condition, (**b**) strain/bridge input module connected to USB data acquisition device, and (**c**) computer reading screen.

The electrical measurement device consists of several devices. The voltage part is measured by the NI-9234 voltage measurement module in the USB data acquisition device of NI cDAQ-9174 and the NI-9237 strain/bridge input module (National Instruments, Austin, TX, USA) to measure the output voltage of the fiber energy collector and the strain at the time of tapping, as shown in Figure 4a. The strain relief/bridge input module includes the CHIEF TB-120 bridge terminal block. The single-axis three-wire strain gauge is connected to the bridge terminal block by a quarter-bridge method, which the strain gauge resistance is about 120 Ω and the gauge factor (GF) is about 2. The measurement can be conducted by attaching the strain gauge to the energy acquisition device, as shown in Figure 4b. The experiment is performed with FleXense software (Chief SI, Hsinchu, Taiwan), and the signal waveforms and values on the computer screen are used to record the voltage data generated during the piezoelectric fiber deformation transients, as shown in Figure 4c. The current is measured with the CH Instruments 611C microelectric flow meter, which has a precision of 10–12 A. Finally, connect the positive and negative terminals of the sample to the input of the instrument for measurement.

## 3. Results and Discussion

**Figure 5:**
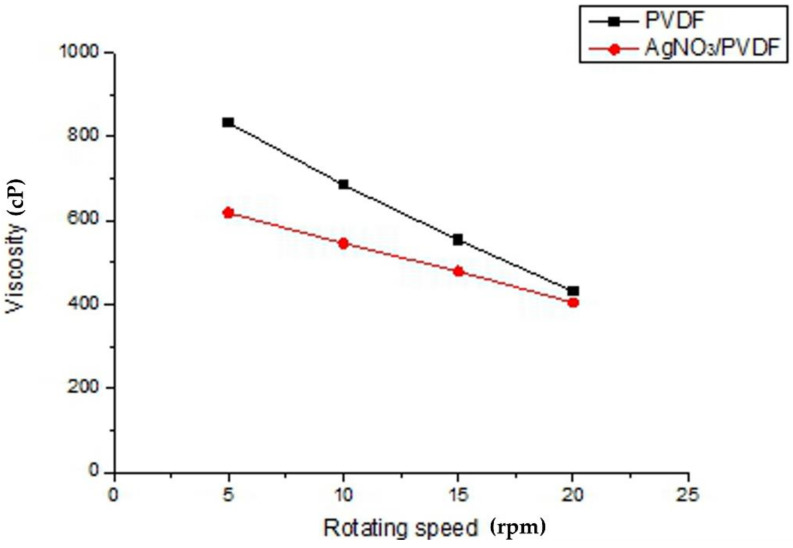
Analysis of solution viscosity characteristics of electrospinning.

The viscosity of the solution is a factor that directly affects the formation of Taylor cones. When the viscosity of the solution is too high or too low, electrospray or discontinuity may occur when an electric field is applied, or the solution may drip directly onto the collection plate and cause a short circuit, resulting in the inability to produce electrospun fibers continuously. Figure 5 represents the solution viscosity characteristics of electrospinning. It can be seen that the viscosity of PVDF solution (18 wt%) decreased from 823.3 cP to 539.5 cP when the rotational speed increased from 5 rpm to 20 rpm during the viscosity value measurement. Further, when the weight percentage of solution was greater than 18 wt%, the viscosity increased and the solution cannot pass through the needle smoothly, resulting in clogging. When AgNO_3_ was added, the parameters of viscosity and intermolecular adhesion were affected by the change in molecular linkage of the electrospinning solution. The viscosity of PVDF solution would be reduced because of the addition of AgNO_3_ solution. This AgNO_3_ solution would dilute the PVDF solution and further reduce the viscosity, which can make the solution more easily break through the surface tension in the electrospinning process. The viscosity dropped from 633.2 cP to 422.7 cP as the speed increased from 5 rpm to 20 rpm. The shear stress of both solutions increased with the increase in speed, while the viscosity decreased with the increase in speed.

**Figure 6:**
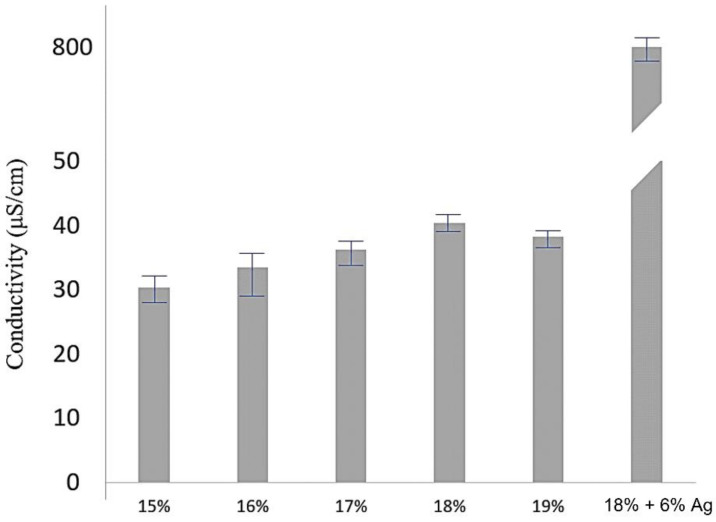
Electrical conductivity of single PVDF electrospinning solutions (15wt%~19wt%) and PVDF electrospinning solution with 6% AgNO_3_.

Figure 6 shows the measurement of electrical conductivity for different weight percentages of PVDF solution and the addition of AgNO_3_ to enhance the electrical conductivity of the electrospinning solution. The results showed that the conductivity of the 18 wt% PVDF solution was the highest at 40.33 μS/cm, which means that it is easier to accumulate charge on the droplets so that the droplets can break through the surface tension and form electrospinning fibers. Furthermore, the electrical conductivity increased from 40.33 μS/cm to 883.59 μS/cm by adding AgNO_3_ to 18 wt% PVDF solution, thus confirming that the electrical conductivity could increase about 20 times by a AgNO_3_.

**Figure 7:**
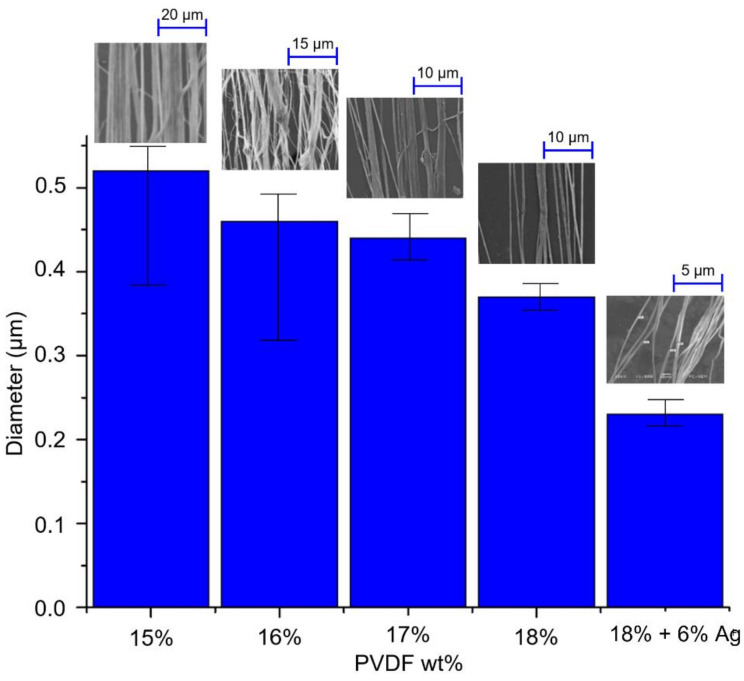
Relationship between electrospinning solution concentration and fiber diameter.

The concentration and conductivity of the solution affect the characteristics of near-field electrostatic spinning fibers. During the experiment, the driving voltage was fixed at 16 kV, the distance between the needle and the collection plate was 1 mm (electric field strength of 1.6 × 10^7^ V/m), the pushing speed of the precision flow control pump was 0.01 mL/min, the inner diameter of the stainless-steel needle was 0.381 mm, and the roller speed was 1500 rpm (tangent speed 1570.7 mm/s). The near-field electrostatic spinning process was carried out with different concentrations of PVDF solution and AgNO_3_.added solution. The results showed that the wire diameter of the fibers in all PVDF solutions was distributed in the range of 0.23–0.52 μm, as shown in Figure 7. During the electrospinning process, when a high voltage is applied and the charge accumulates to a limit value, the liquid will break through the surface tension and shoot out droplets to form Taylor cone. In the 15 wt% and 16 wt% electrospinning solutions, the bonding force between the liquid molecules is not sufficient, which results in discontinuous fiber formation during the droplet formation process, thus causing a large error in the diameter of the fiber. When 18 wt% PVDF solution was used, the minimum fiber diameter can reach to 0.37 μm. From the results, it can be seen that the higher the weight percentage, the smaller the PVDF piezoelectric fiber wire diameter tends to be. The reason for this is that the conductivity of 18 wt% PVDF solution is higher than other concentrations, which makes it easier for the droplets to break through the surface tension and form Taylor cones. Due to the higher conductivity of the electrospinning solution, the resistance of the electrospinning process is lower and therefore the fiber diameter is smaller. Furthermore, with the addition of AgNO_3_, the wire diameter can be reduced to 0.23 μm, which is about 30% lower than the wire diameter of the electrospinning solution without the addition of AgNO_3_.

**Figure 8:**
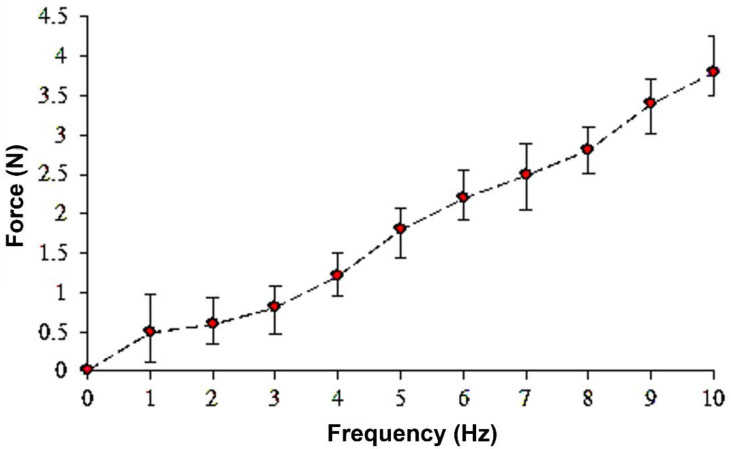
Force analysis of PVDF piezoelectric fiber energy acquisition device when applying a tapping frequency of 1–10 Hz.

PVDF piezoelectric fibers are placed on a parallel electrode energy acquisition device and tapped at a frequency of 1–10 Hz. The relationship between the frequency of the rotary tapper beat and the power reading is measured using a load meter. The results showed that the energy acquisition device of PVDF piezoelectric fiber applied a tapping frequency of 1–10 Hz with a corresponding force of 0.5~4 N, as shown in Figure 8. When the tapping frequency is higher, the higher the impact frequency provided and the higher the force required. In addition, for the voltage measurement, the tapping frequency of 4–7 Hz was selected because the output voltage signal was not obvious at low tapping frequency, while the voltage output signal was obvious at 4–7 Hz.

**Figure 9:**
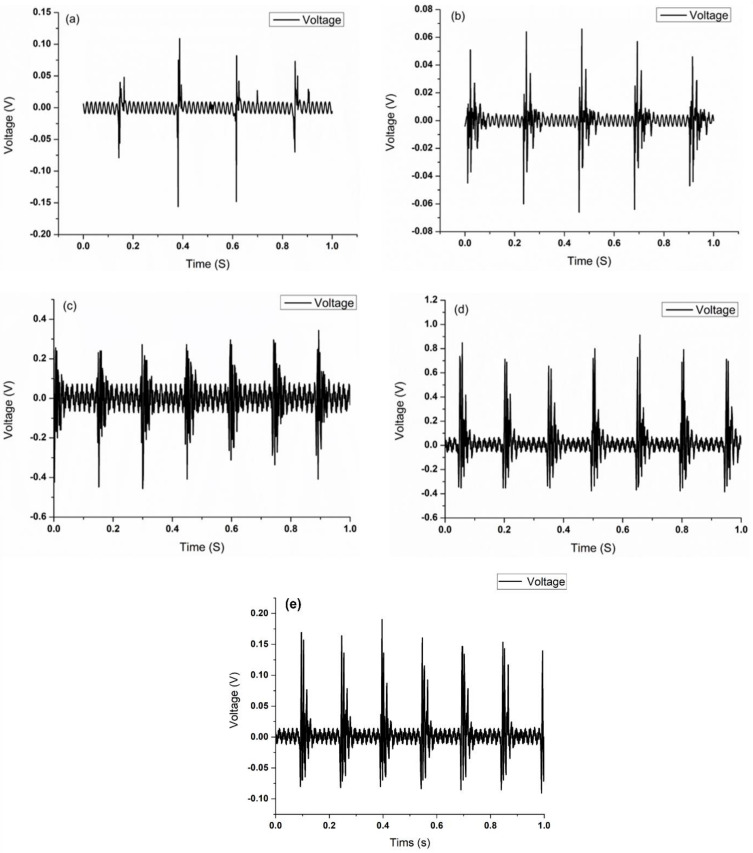
Piezoelectric properties (voltage output) of PVDF fibers with 6% Ag nanoparticles: tapped (**a**) at 4 Hz; (**b**) at 5 Hz; (**c**) at 6 Hz, (**d**) at 7 Hz, and (**e**) without Ag nanoparticles.

Different electrospun fibers were tested for their piezoelectric properties to measure the voltage released by the transient deformation. Different fibers were packaged into electrical measurement devices by PDMS, and the fibers were beaten by a rotary tapper to produce transient deformation and measured the resulting electrical properties. For the electrospun solution configured with 18 wt% PVDF powder, the average maximum output voltage is ~0.15 V at a strain of 0.027, while the average maximum output voltage of the electrospun fiber with the addition of AgNO_3_ (6%) increased to ~0.9 V at a frequency of 7 Hz (strain of 0.027), which is more than four times of the single PVDF fiber, as shown in Figure 9. The result proves that the increase in electrical conductivity of the material itself improves the overall piezoelectric properties of the fiber.

**Figure 10:**
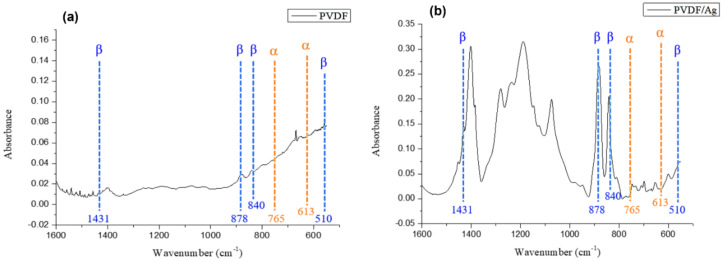
FTIR results of (**a**) 18 wt% of single PVDF and (**b**) with the addition of 6% Ag nanoparticles electrospinning solution after polarization.

Most of the unpolarized PVDF powder exhibits α-phase crystallization, and the dipole moments are randomly arranged and cancel each other, so PVDF does not have piezoelectricity. The structure of the material after electrospinning are shown in Figure 10. In Figure 10a, it can be seen that the α-phase (613 cm^−1^, 765 cm^−1^) decreased after polarization of the electrospun solution with a single PVDF powder, while the β-phase (510 cm^−1^, 840 cm^−1^, 878 cm^−1^, and 1431 cm^−1^) with piezoelectric properties increased. As a result, when PVDF was polarized, its internal dipole moment would rearrange, and the original disorderly arrangement would convert into a single direction, so that the internal α-phase of the material would change to β-phase. Furthermore, when AgNO_3_ were added, the Ag nanoparticles interacted with PVDF at the interface and affect the dispersion of the Ag nanoparticles in the polymer matrix and the dielectric properties of the composite material. In Figure 10b, it is found that the added Ag nanoparticles still had the α, β and γ phases of PVDF after electrospinning, where the α phase decreased and the β phase increased, indicating that the Ag nanoparticles could induce the increase of β phase of PVDF. With the addition of 6% AgNO_3_, Ag ion was attracted to the –CF_2_– dipole of PVDF and repelled by the –CH_2_– dipole of PVDF [26]. The interaction of attractive and repulsive forces as well as the enhanced interfacial polarization promoted the generation of β-phase in PVDF, as can be seen in Figure 10. Further, the conductivity of PVDF solution increased and correspondingly the stretching in the electric field was enhanced, which further generated more β-phase. In addition to α, β peaks, peaks between 1000 cm^−1^ and 1300 cm^−1^ correspond to –CH_2_ groups in the polymer backbone and –CF_3_ stretching vibration. These peaks are larger in PVDF/AgNO_3_ compared to that of in PVDF, implying an enhanced effect of the addition of AgNO_3_. However, no Ag peak was observed in the FTIR result since Ag nanoparticles are not sensitive to the IR spectra [27].

**Figure 11:**
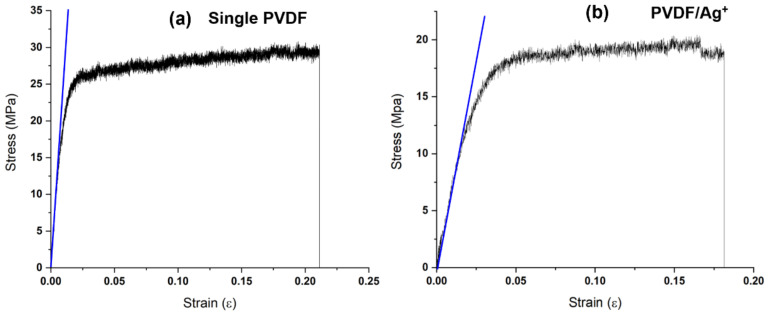
Stress-strain curves of the as prepared fibers: (**a**) single PVDF and (**b**) PVDF/Ag.

The stress-strain curves of the as prepared fibers are shown in Figure 11. It was found that when AgNO_3_ was added to PVDF electrospinning solution, the tensile strength of the fibers decreased from ~25 MP to ~19 MPa due to the defects in the material of the fibers during the electrospinning caused by Ag ions. However, the application in this study did not cause any effect.

**Figure 12:**
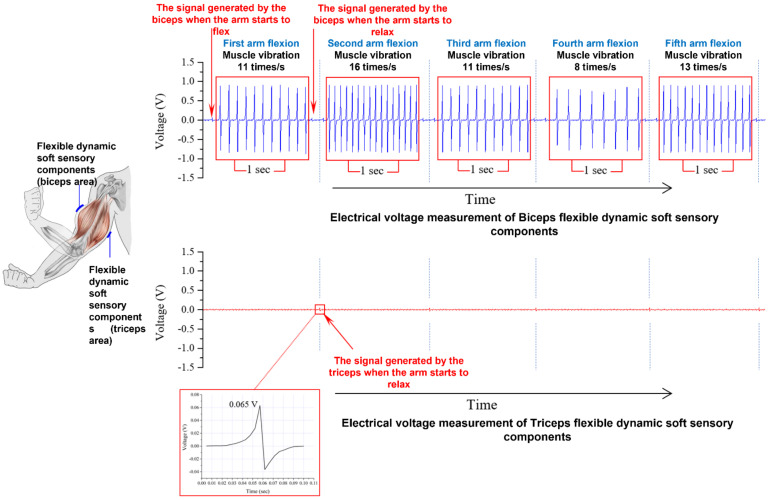
Data results of the trembling phenomenon when the human body continues to hold the dumbbell during a long period of exercise under a load that has exceeded the human body’s muscle strength.

In the study, the PVDF piezoelectric fibers were effectively enhanced by the addition of AgNO_3_ nanoparticles, and the flexible dynamic soft-sensitive elements were used to detect muscle tremors after high-intensity training, allowing for a more accurate diagnosis and treatment of fasciculation. Through hand muscle movement (dumbbell lifting action), the flexible dynamic soft sensing component was attached to the arm muscle, as shown in Figure 12. In the study, the loading of the arm muscle was lifted by the weight of the dumbbell, and the relationship between the muscle tremor and the voltage output of the flexible dynamic soft sensing component was observed. When the human body was exercising for a long period of time under a load that exceeds the human body’s muscle strength, the biceps muscle was found to produce 8 to 16 tremors per second through five arm flexion movements, and the voltage output of the flexible dynamic soft sensor was between 0.7 and 0.9 V. The AC wave pattern with an orderly voltage signal was observed. Furthermore, when the arm was flexed, the biceps muscle generated an initial signal, and although this voltage signal was only ~0.06 V output, the difference between tremor and muscle protrusion could be confirmed. The same muscle protrusion signal is also present in the flexible dynamic soft sensory detection element attached to the triceps brachii muscle. When the arm is lowered at the end of the arm flexion movement, the dumbbell is also in the hand, and at this time there would be a muscle protrusion phenomenon in the triceps brachii muscle.

## 4. Conclusions

PVDF and AgNO_3_/PVDF composite piezoelectric fibers were prepared using near-field electrospinning technology. The prepared fibers were attached to the electrode sheet and encapsulated with PDMS and further fabricated into a dynamic sensing element. The conductivity results showed that the solution of single PVDF with 18 wt% was the highest with 40.33 μS/cm, while the conductivity of the AgNO_3_/PVDF composite solution increased to 883.59 μS/cm. When the AgNO_3_/PVDF mixed solution droplet was subjected to a high voltage field of 1.6 × 10^7^ V/m, a piezoelectric fiber with a fiber diameter of 0.23 μm was obtained, which was smaller than that of a single PVDF with a fiber diameter of 0.37 μm, indicating that the addition of Ag nanoparticles helped to reduce the fiber diameter size. The FTIR results showed that the high field polarization of PVDF and AgNO_3_/PVDF composite solution can lower the α-phase PVDF and increase the β-phase PVDF after NFES process, and the addition of Ag nanoparticles can further effectively increase the β-phase and increase the voltage value that can be read by the energy acquisition device. The average maximum output voltage of single PVDF was found to be 0.15 V, while the average maximum output voltage of the composite piezoelectric material after adding Ag nanoparticles can be increased to 0.9 V. When the human body is loaded beyond the muscular force and the arm is flexed five times, the biceps muscle is found to produce 8 to 16 tremors per second, and the voltage output of the flexible dynamic soft sensing element is between 0.7 and 0.9 V. Although the sensor in the study is flexible, there is still a problem of damage to the package structure under high bending. The reliability of the sensor will be further investigated in the future. The PVDF/AgNO_3_ composite piezoelectric fiber made by near-field electrostatic spinning has the characteristics of good ductility of PVDF polymer and high piezoelectricity of AgNO_3_, which can increase the versatility of application.

## Figures and Tables

**Table 1:** Preparation of pure PVDF.

Solution A		Solution B	
PVDF (g)	Acetone (g)	DMSO (g)	Surfactant (g)
0.9	2.5	0.2	2.5

## Data Availability

Not applicable.
